# A Fungal Symbiont of Plant-Roots Modulates Mycotoxin Gene Expression in the Pathogen *Fusarium sambucinum*

**DOI:** 10.1371/journal.pone.0017990

**Published:** 2011-03-24

**Authors:** Youssef Ismail, Susan McCormick, Mohamed Hijri

**Affiliations:** 1 Institut de recherche en biologie végétale (IRBV), Département de sciences biologiques, Université de Montréal, Montreal, Quebec, Canada; 2 Plant Pathology Unit, Department of Plant Protection, Desert Research Center, Cairo, Egypt; 3 Bacterial Foodborne Pathogens and Mycology Research Unit, NCAUR, U.S. Department of Agriculture, Peoria, Illinois, United States of America; University of Wisconsin-Milwaukee, United States of America

## Abstract

*Fusarium* trichothecenes are fungal toxins that cause disease on infected plants and, more importantly, health problems for humans and animals that consume infected fruits or vegetables. Unfortunately, there are few methods for controlling mycotoxin production by fungal pathogens. In this study, we isolated and characterized sixteen *Fusarium* strains from naturally infected potato plants in the field. Pathogenicity tests were carried out in the greenhouse to evaluate the virulence of the strains on potato plants as well as their trichothecene production capacity, and the most aggressive strain was selected for further studies. This strain, identified as *F. sambucinum*, was used to determine if trichothecene gene expression was affected by the symbiotic Arbuscular mycorrhizal fungus (AMF) *Glomus irregulare*. AMF form symbioses with plant roots, in particular by improving their mineral nutrient uptake and protecting plants against soil-borne pathogens. We found that that *G. irregulare* significantly inhibits *F. sambucinum* growth. We also found, using RT-PCR assays to assess the relative expression of trichothecene genes, that in the presence of the AMF *G. irregulare*, *F. sambucinum* genes *TRI5* and *TRI6* were up-regulated, while *TRI4*, *TRI13* and *TRI101* were down-regulated. We conclude that AMF can modulate mycotoxin gene expression by a plant fungal pathogen. This previously undescribed effect may be an important mechanism for biological control and has fascinating implications for advancing our knowledge of plant-microbe interactions and controlling plant pathogens.

## Introduction

Secondary metabolites are compounds produced by many organisms including filamentous fungi. These compounds include pigments, toxins, plant growth regulators, antibiotics and numerous compounds used for pharmaceutical purposes [1]. Many fungal secondary metabolites increase the fitness of the organisms toward in adverse environmental conditions [2], [3]. The fungal genus *Fusarium* (teleomorph: *Giberrella*) consists of over 70 species, many of which are plant pathogens, and some species can produce secondary metabolites, known as mycotoxins, that are toxic to humans and animals [1], [4]. Trichothecenes are a family of terpene-derived mycotoxins that impact human, animal [5] and plant [6].

Trichothecene biosynthetic gene clusters have been characterized in *F. graminearum* and *F. sporotrichioides*, [1], [7]. In both species, there is a core cluster of 12 genes that are involved in the biosynthesis, regulation or transport of trichothecenes. These genes are: *TRI5* (encoding a terpene synthase); *TRI4*, *TRI11* and *TRI13* (encoding cytochrome P450 monooxygenases); *TRI3* and *TRI7* (encoding acetyl transferases), *TRI8* (encoding an esterase), *TRI6* and *TRI10* (proposed to be regulatory genes) and *TRI12* (encoding a transporter). In addition, there are also two genes, *TRI9* and *TRI14* with unknown functions. The biosynthetic pathway for all trichothecenes begins with a cyclization of farnesyl pyrophosphate to produce the hydrocarbon trichodiene [8]. Trichodiene is then converted to highly toxic molecules such as 4,15-diacetoxyscirpenol (DAS), the predominant mycotoxin of *F. sambucinum*, through a series of oxygenation, isomerization, cyclization, esterification, and deacetylation steps (Figure S1).

Control strategies against fungal pathogens that produce mycotoxins are mainly based on the use of fungicides [9], although biological-control agents [10] and plant-resistant varieties have been reported. However, the use of AMF to control mycotoxin-producing microorganisms has not been previously reported. AMF inhabit plant roots and not only form a symbiotic association with most plant species but also interact with a wide range of other soil organisms such as soil bacteria [11]. AMF are well known to promote plant growth and are largely used as commercial inoculants as bio-fertilizers worldwide. Many literature reports showed that AMF can reduce the incidence and severity of root diseases and protect plants against soil-borne pathogens [12]. However, the mechanisms by which AMF may act as biological control agents are not known. Three mechanisms have been hypothesized: soil microbial community changes, antagonisms and stimulation of plant defenses. AMF can inhibit or promote soil microorganisms [11]. It has been reported that soluble substances released by the extraradical mycelium of *Glomus intraradices* grown *in vitro*, stimulated both the growth of *Pseudomonas chlororaphis* and the germination of *Trichoderma harizianum* conidia. In contrast, the germination of *Fusarium oxysporum* f. sp. *chrysanthemi* conidia was reduced in the presence of the AMF extract [13].

In this study, we identified 16 isolates belonging to the genus *Fusarium* from naturally infected potato plants and confirmed that nine produced trichothecenes. One isolate, *F. sambucinum* strain T5 induced a rapid wilting and yellowing that resulted in plant death and was selected for further studies. The objective of this work was to assess the effect of the AMF *G. irregulare* on growth and expression of *TRI* genes by *F. sambucinum*. We addressed three specific points: *i)* trichothecene production by *F. sambucinum* using molecular and chemical approaches; ii) effect of the AMF *G. irregulare* on the growth of trichothecene-producing *F. sambucinum in vitro*; and iii) *G. irregulare-*induced modulation of trichothecene gene expression in *F. sambucinum*.

## Results and Discussion

### Characterization of *TRI* genes of *F. sambucinum*

**Figure 1:**
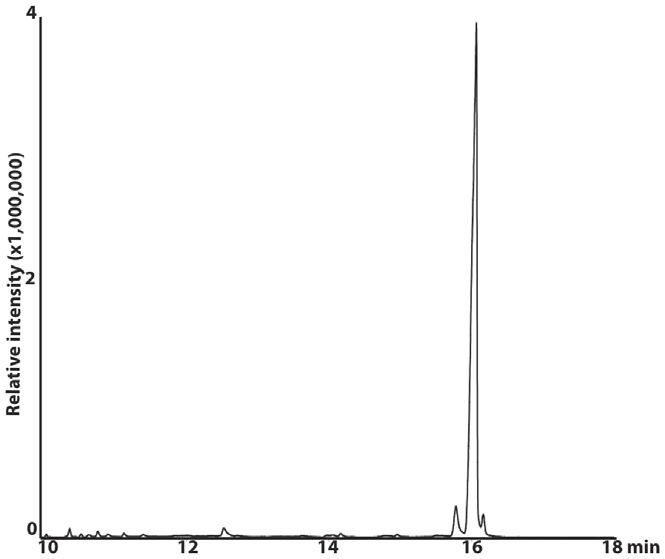
GC-MS traces of Diacetoxyscirpenol (DAS). Reconstructed ion chromatogram of an extract of 7 day-old liquid stage 2 cultures of *F. sambucinum* (T5). DAS elutes at 16.1 minutes.

**Figure 2:**
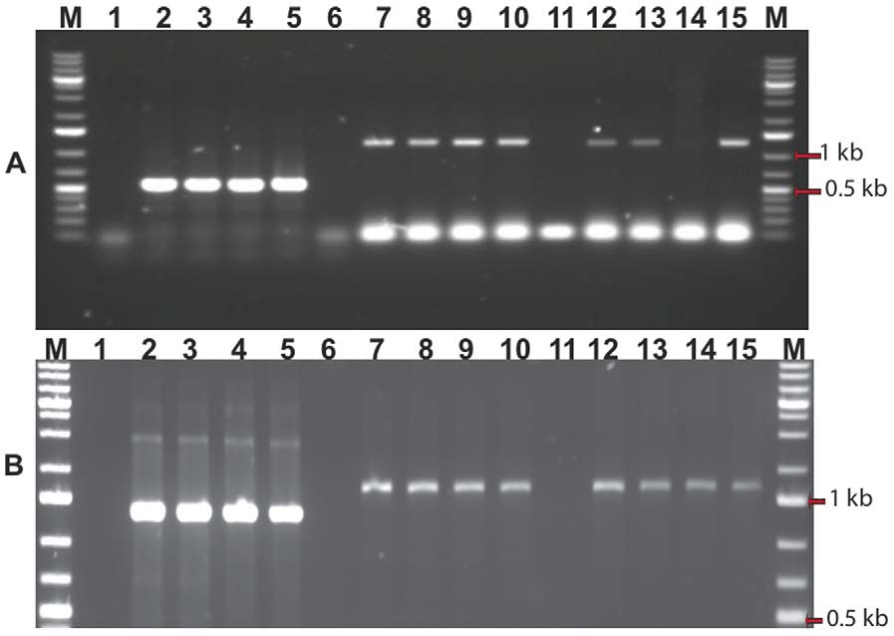
PCR amplification of ITS and *TRI* genes. **Panel A:** Agarose gel electrophoresis showing PCR products of ITS and *TRI* from four *F. sambucinum* strains T3, T5, T6 and T8 isolated from naturally infected potato. Lanes (2–5) show ITS PCR products from strains T3, T5, T6 and T8 respectively. Lanes (7–10) and (12–15) show *TRI5* and *TRI101* PCR products from T3, T5, T6 and T8 respectively. Lanes, 1, 6 and 11 are negative controls. Lane M, shows 1 Kb ladder. The PCR products for *TRI4* (1.4 kb; lanes 7–10) and for *TRI11* (1.5 kb; lanes 12–15) are of the expected size. **Panel B:** shows PCR patterns of *TRI3* (lanes 2–5), *TRI4* (lanes 7–10 and 12–15) from strains T3, T5, T6 and T8, respectively. Lanes, 1, 6 and 11 are negative controls. Lane M shows 1 kb ladder.

We isolated sixteen strains of *Fusarium* from roots and tubers of naturally infected potato plants, and found that nine of these produced trichothecenes. We chose *F. sambucinum* strain T5 as a model for this study because it was the most aggressive strain when tested on potato plants, inducing a rapid wilting and yellowing that resulted in plant death (Figure S2). This strain produced 4,15-diacetoxyscirpenol (DAS) when grown in liquid culture (Figure 1). We used ITS regions of ribosomal rRNA genes and morphology to confirm its identity. Nucleotide BLAST search showed 100% sequence identity with *Gibberella pulicaris* strain NBAIM: 455 (anamorph: *F. sambucinum*). This strain is a causal agent of dry rot of tuber crops [14]. We used degenerate primers (Table 1) and DNA from *F. sambucinum* to amplify fragments corresponding to five trichothecene genes (*TRI5*, *TRI4*, *TRI101*, *TRI3*, and *TRI11*) involved in production of DAS (Figure 2). PCR gave bands of the expected sizes ranging from 1.1 to 1.5 Kb. These PCR products were sequenced and, when compared to the NCBI database using nucleotide BLAST searches, showed 98% and 97% sequence identity with trichothecene biosynthetic genes of *F. sporotrichioides* and *Gibberella zeae*, respectively.

**Table 1:** Primers sets used for PCR and RT-PCR experiments.

Primer	Nucleotide sequences (5′-3′)	Target gene	Reaction
ITS1	TCCGTAGGTGAACCTGCGG	*ITS*	PCR
ITS4	TCCTCCGCTTATTGATATGC	*ITS*	PCR
1912	TGTGTMGGYGCWGAGGCVATYGTTGG	*TRI3*	PCR
1914	ACRGCAGCRGTCTGRCACATGGCGTA	*TRI3*	PCR
1450	ACCTTGAGTTCTACCATGAAGTCATC	*TRI4*	PCR
1455	GCACTGTCTAGARCCCTGAGAGAAGT	*TRI4*	PCR
1558	GGCATGGTCGTGTACTCTTGGGTCAAGGT	*TRI5*	PCR
1559	GCCTGMYCAWAGAAYTTGCRGAACTT	*TRI5*	PCR
1482	CACACYCTCCTSATGCTYTGTGGACT	*TRI11*	PCR
1483	TCCCAMACTGTYCTYGCCAGCATCAT	*TRI11*	PCR
109	CCATGGGTCGCRGGCCARGTSAA	*TRI101*	PCR
178	AACTCSCCRTCIGGYTTYTTNGGCAT	*TRI101*	PCR
ß-tubulin-F	GCCATGAAAGGAGGTTGAGGA	*Ref. gene*	RT-PCR
ß-tubulin-R	AAGCCTTGCGTCGGAACATA	*Ref. gene*	RT-PCR
EF 1α-F	GTACGCCTGGGTTCTTGACA	*Ref. gene*	RT-PCR
EF 1α-R	GAGCGTCTGGTAGGCATGTTAG	*Ref. gene*	RT-PCR
TRI4-F	GCCACTGCTGCTACTGTTG	*TRI4*	RT-PCR
TRI4-R	GGTCGTTGTCCAGATGTTCTTG	*TRI4*	RT-PCR
TRI5-F	TGGAGAACTGGATGGTCTGG	*TRI5*	RT-PCR
TRI5-R	GACATAGCCGTGCATGAAGC	*TRI5*	RT-PCR
TRI6-F	AGTGCCAAGTCAGCTCATCG	*TRI6*	RT-PCR
TRI6-R	GAGCACGATCCTTGCGAGTT	*TRI6*	RT-PCR
TRI13-F	CTGCGGTGGAACCGCTGGTA	*TRI13*	RT-PCR
TRI13-R	ACACTGGCGTTGTCCGTAAG	*TRI13*	RT-PCR
TRI101-F	ATCGCCAACGAACCACTTG	*TRI101*	RT-PCR
TRI101-R	TGATGCTGCTTGACGGATTC	*TRI101*	RT-PCR

**Figure 3:**
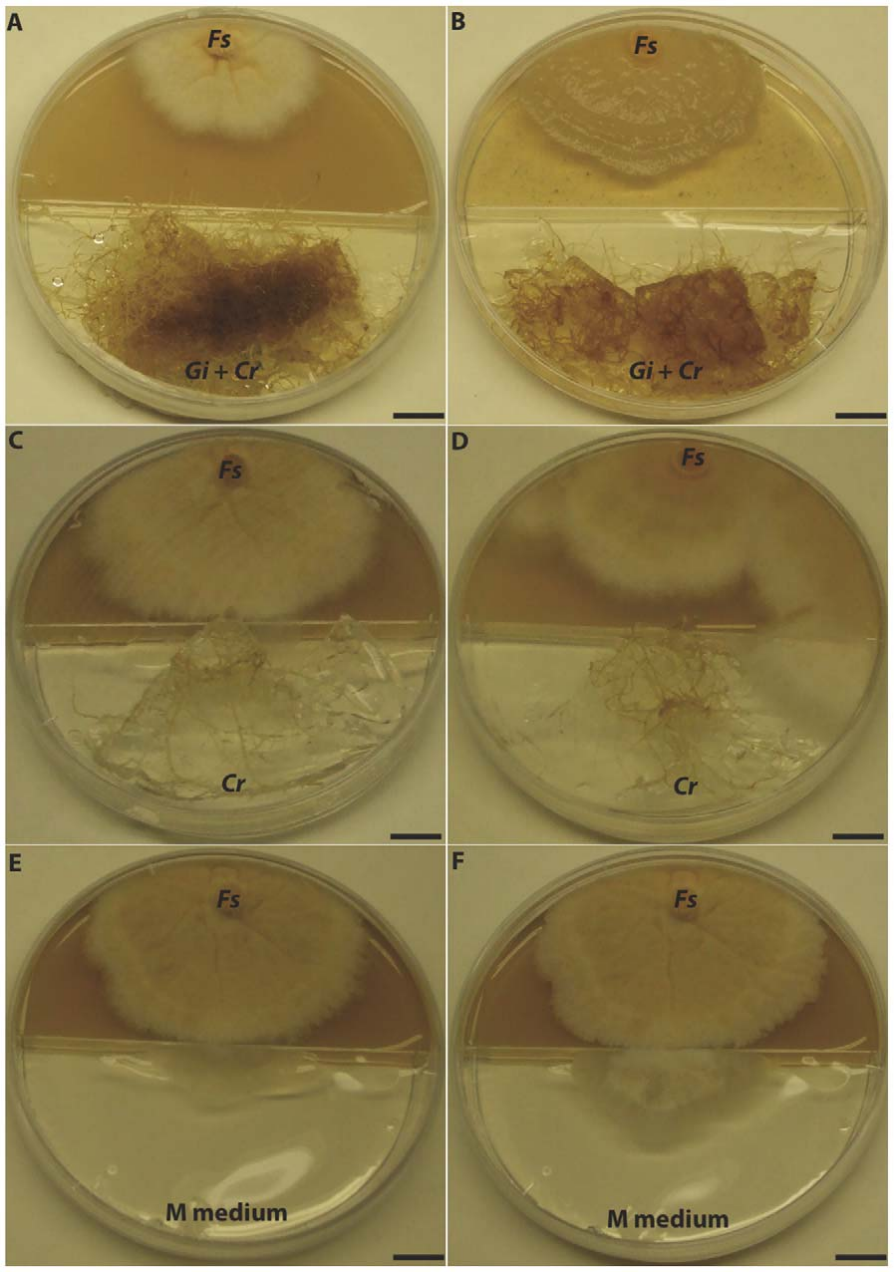
Confrontation cultures between *G. irregulare* and *F. sambucinum*. Experiments of confrontation between *F. sambucinum* and 2 isolates of *G. irregulare* DOAM-197198 (A) and DOAM-234328 (B) were performed in two-compartment Petri plates. Controls consisted of carrot roots without AMF (C & D) and M medium (E391 & F) without carrot roots or AMF.

To study the impact of *G. irregulare* on the growth of *F. sambucinum*, we used confrontation cultures using an *in vitro* system. We found that the growth of *F. sambucinum* was significantly reduced in the presence of *G. irregulare* isolate DAOM-197198 compared with controls that consisted of carrot roots without *G. irregulare* or *F. sambucinum* alone (Table 2). *G. irregulare* significantly reduced the growth of *F. sambucinum* after 3, 5, 7 and 15 days (Figure 3). Interestingly, inhibition of the growth *F. sambucinum* was associated with morphological changes in the presence of *G. irregulare* compared with controls (Figure 3). These changes were observed when *G. irregulare* hyphae reached *F. sambucinum* mycelium, suggesting that *G. irregular* may produce compounds that interfere with normal the growth of *F. sambucinum*.

**Table 2:** Effect of *G. irregulare* on growth of *F. sambucinum*.

	Fungal growth (cm^2^)							
Treatment	3-days		5-days		7-days		15-days	
	Growth*	SD**	Growth	SD	Growth	SD	Growth	SD
Fs+M medium	**14.50** ^a^	**1.42**	**25.62** ^a^	**3.26**	**29.71** ^a^	**3.73**	**42.69** ^a^	**4.18**
Fs+Carrot roots	**15.79** ^a^	**2.02**	**25.30** ^a^	**3.87**	**28.74** ^a^	**2.92**	**46.04** ^a^	**4.33**
Fs+DAOM-197198	**7.55** ^b^	**2.15**	**9.31** ^b^	**1.67**	**11.40** ^b^	**1.42**	**12.63** ^b^	**1.54**

*Values are means of 20 replicates. Within each column values followed by the same letter are not significantly different using one-way ANOVA analysis.

**Standard deviation of the mean.

Expression of *TRI4, TRI5, TRI6, TRI13 and TRI101 genes*.

**Figure 4:**
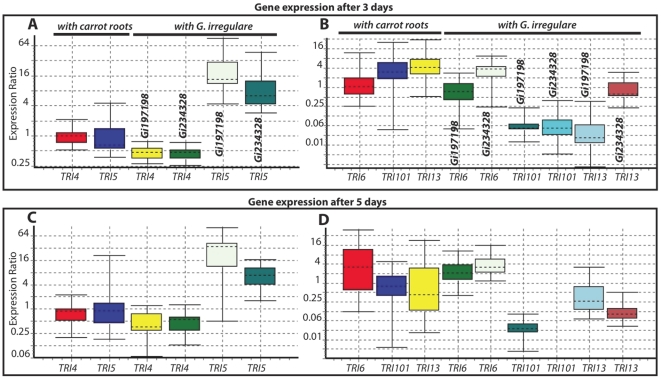
Relative expression patterns of *TRI4, TRI5, TRI6, TRI101* and *TRI13* genes from *F. sambucinum* compared with two reference genes ß-tubulin and EF-1α. Relative expression patterns of *TRI* genes of *F. sambucinum* after 3 days (A and B) and 5 days (C and D) of confrontation either with carrot roots lacking AMF, or with *G. irregulare* colonized carrot roots. Panels A and C show relative expression patterns of *TRI4* and *TRI5* for *F. sambucinum* confronted with carrot roots and against *G. irregulare* isolates DAOM-197198 (Gi197198) and DAOM-234328 (Gi234328), respectively. Panels B and D show relative expression patterns of *TRI6*, *TRI101* and *TRI13* of *F. sambucinum* against carrot roots and *G. irregulare* isolates DAOM-197198 (Gi197198) and DAOM-234328 (Gi234328), resperctively.

To test whether *G. irregulare* modulates the expression of *TRI* genes, we carried out real-time qRT-PCR assays on *TRI4* and *TRI5* on total RNA extracted from *F. sambucinum* grown alone or confronted with *G. irregulare* isolates DAOM-197198 and DAOM-234328 during 3 and 5 days. *TRI4* and *TRI5* genes encode P450 oxygenases and trichodiene, respectively [15]. We used ß-tubulin and the translation factor EF1α as reference genes for RT-PCR assays. Figure 4 shows relative expression patterns of *TRI4* and *TRI5* genes when *F. sambucinum* was confronted with the two isolates of *G. irregulare*. Interestingly, the relative expression of *TRI5* in *F. sambucinum* was up-regulated (*p<0.001*) by a factor of 17 and 8 after 3 days of confrontation with *G. irregulare* isolates DAOM-197198 and DAOM-234328, respectively. *TRI5* encodes a trichodiene synthase and is the first enzyme involved in the trichothecene biosynthesis pathway [1]. In contrast, the relative expression of *TRI4* (encoding a multifunctional P450 oxygenase) was down-regulated (*p<0.003*) by a factor of 0.46 and 0.43 when confronted with isolates DAOM-197198 and DAOM-234328, respectively. Relative expression of *TRI5* and *TRI4* genes was not affected (*p>0.22*) when *F. sambucinum* was grown alone or confronted with carrot root without *G. irregulare* (Figures 4A; 4C). We used carrot root as a control because *G. irregulare* is an obligate biotroph that requires plant roots for its culture. These controls clearly show that over-expression of *TRI5* and down-regulation of *TRI4* was due to *G. irregulare*.

Interestingly, the two isolates of *G. irregulare* showed different modulation levels on the expression of *TRI5* and *TRI4* genes in *F. sambucinum*. This difference in the response of *G. irregulare* isolates could be explained by their genetic composition. It is well documented in the literature that AMF have a high intra- and inter-isolate genetic diversity [16], [17], [18]. Recently, Angelard and coworkers [19] showed that segregated lines of the AMF *G. irregulare* (formerly *G. intraradices*) obtained from crossing two genetically different parents have a huge effect on host-plant response [19].

To test the effect of *G. irregulare* on other trichothecene biosynthetic genes, we carried out additional RT-PCR assays on *TRI6*, *TRI13*, and *TRI101*, using an experimental setup similar to that used for *TRI4* and *TRI5* (Fig. 4B and D). Relative expression of the transcription factor *TRI6* gene was only up-regulated (*p<0.03*) by a factor of 2.8 and 3.6, after 3 and 5 days of confrontation with *G. irregulare* DOAM-*234328*, respectively (Fig. 4B and D). Surprisingly, isolate DOAM-197198 produced no significant effect (*p>0.08*) on relative expression of *TRI6* from *F. sambucinum*. This finding strongly supports genetic heterogeneity and phenotypic differences among isolates of *G. irregulare*. *TRI6* encodes a zinc finger protein involved in regulation of trichothecene biosynthesis [20] with expression of *TRI5* and *TRI4* genes dramatically reduced or silenced in *TRI6* disruption mutants [21]. Relative expression patterns of *TRI13* and *TRI101* were greatly down-regulated (*p<0.001*) after 3 and 5 days of confrontation with *G. irregulare* isolates, respectively (Fig. 4B and D). *TRI101* encodes a trichothecene 3-O-acetyltransferase that acetylates the C-3 of various *Fusarium* trichothecenes, converting them to less toxic products [22], [23]. In biosynthesis of DAS, the *TRI101* acetyltransferase catalyzes the conversion of isotrichodermol to isotrichodermin [22], [23].

Control of mycotoxin-producing fungal pathogens has largely relied on the use of chemicals [9]. Modulation of trichothecene biosynthetic gene expression by AMF may be a safer way of limiting mycotoxin production. The impact of AMF on plant pathogenic fungi has been studied under ecological conditions and in a large number of host-pathogen interactions [12], [24]. These interactions can be direct, such as competition with the pathogen, or indirect, including (1) alleviation of abiotic stress such as enhanced nutrition of the host plant, (2) biochemical induced changes, and (3) interactions with microbiota in the rhizosphere . Most of the direct effects have been a result of AMF interacting with pathogens in the rhizosphere in which complex associations exist among plant roots, soil, and microorganisms [11]. However, changes in plant root physiology due to AMF association are certain to have significant impacts on the rhizosphere microflora through alteration of root exudates and other nutrient-related mechanisms [12], [24]. A direct interaction between *G. irregulare* and *F. oxysporum* has been studied with axenic system designed by St-Arnaud et al., (1995) [25] in which *G. irregulare* altered the growth of *F. oxysporum*. In this study, we also showed that *G. irregulare* had a significant inhibitory effect on growth of a virulent and mycotoxin-producing isolate of *F. sambucinum*. In addition, *G. irregulare* significantly down-regulated three trichothecene biosynthetic genes, *TRI4, TRI13, TRI101*. Confrontation with *G. irregularre* increased the expression of the trichodiene synthase gene *TRI5*, which may be a response to the down-regulation of the P450 oxygenase gene *TRI4*. Up-regulation of *TRI6*, a *G. irregulare* regulatory gene in trichothecene biosynthesis, may also contribute to the increase in TRI5 expression [26]. Our study confirms and demonstrates AMF influence on the growth of *F. sambucinum* and furthermore have an effect on mycotoxin biosynthetic gene expression. Further chemical analyses will be required to see if the changes observed in trichothecene gene expression in dual confrontation result in a change in trichothecene production. It has been reported that signaling volatile organic compounds known as semiochemicals have effects in plant protection [27]. For example, the production of these semiochemicals by plant tissue can be induced by fungal infection [28]. The production of some semiochemicals directly by some fungi and bacteria was also shown, as well as the possibility of transformed roots to produce semiochemicals [29]. Thus, the effect of *G. irregulare* on gene expression in *F. Sambucinum* could result from a direct effect of the AMF, but also from an indirect effect. *G. irregulare* could induce carrot roots to produce volatiles with activity against trichothecene production in *F. Sambucinum*.

We conclude that AMF can modulate mycotoxin gene expression of a plant fungal pathogen. This effect may be an important mechanism involved in biological control of plant pathogens. This finding brings a new level of understanding to plant-microbe interactions and control of plant pathogenesis.

## Materials and Methods

### Fungal strain and growth conditions

*Fusarium sambucinum* strain T5 was isolated and characterized from naturally infected potato plants (cultivar Riba) from a field located in the Montreal region in August 2008. The strain was grown and maintained on V-8 juice agar medium and in GYEP medium (2% glucose, 0.1% yeast extract, 0.1% peptone) [30]. Two isolates of the AMF *G. irregulare* DAOM-197198 and DAOM-234328 were grown *in vitro* in co-culture with Ri T-DNA-transformed carrot roots (*Daucus carota* L.) on a minimal (M) medium. Spores for both isolates of *G. irregulare* were collected from plates by dissolving the Gellan gum as described [31].

### DNA extraction, PCR amplification and sequencing

DNA was extracted from freshly harvested fungal mycelium grown in liquid GYEP medium for 2–4 days. Fungal mycelium was dried and ground using mortar and pestle and DNA was extracted with DNeasy Plant Mini Kit (Qiagen, Canada) following the manufacturer's instructions. PCR amplifications of trichothecene genes: *TRI1*, *TRI3*, *TRI4*, *TRI4*, *TRI6*, and *TRI101* were performed on *G. pulicaris* (anamorph: *F. sambucinum*) strain T5 using the primer sets listed (Table 1). PCR amplifications in 50 µL all contained: 1× *Taq* buffer, 0.25 mM of each dNTP, 0.5 µM of each primer, 1 U of *Taq* DNA polymerase (Fermentas), and 50 ng of DNA template. Reactions were carried out using a thermal cycler EP Mastercycler S (Eppendorf) under the following parameters: pre-denatured at 94°C for 1 min, followed by 30 cycles of denaturation at 94°C for 30 sec, annealing at 51°C for 30 sec and elongation at 72°C for 90 sec, and a final, elongation at 72°C for 5 min. For each trichothecene gene, a negative control without DNA template was performed. PCR amplificons were visualized on a 1% agarose gel stained with ethidium bromide and visualized under UV light. Reactions that showed clear amplification bands were sequenced at the Genome Quebec Innovation Center (Montreal, Qc), using the specific primers (Table 1). Two sequencing reactions were performed for each PCR amplicon. Recovered sequences were assembled and analyzed using Vector NTI software (Invitrogen) and compared to the NCBI database using Nucleotide BLAST search. Nucleotide sequences were deposited in the EMBL nucleotide sequence database under the accession numbers: HQ445900 for ITS and HQ445905 to HQ445907 for *TRI* genes.

### Chemical analysis of the trichothecenes

To induce mycotoxin production in liquid culture, a two-stage medium protocol modified from the method of Miller and Blackwell (1986) was employed [32], [33]. The cultures were grown at 25°C on a rotary shaker at 200 rpm in the dark. After 7 days of incubation in the second stage medium, a 5 ml aliquot containing fungal material was extracted with 2 ml ethyl acetate. Extracts were analyzed with gas chromatography and low resolution mass spectrometry (GCMS) using a Hewlett Packard 6890 gas chromatograph fitted with a HP-5MS column (30 m×0.25 mm film thickness) and a 5973 mass detector. Trichothecenes were identified by comparison of retention time and mass spectra with standard compounds.

### Dual culture assays

The confrontation cultures between *G. irregulare* and *F. sambucinum* were performed *in vitro* using two-compartment Petri dishes (100×15 mm). One compartment was filled with 25 ml GYEP agar medium (2% glucose, 0.1% yeast extract, 0.1% peptone and 2% agar) for *F. sambucinum*. The other compartment of the plates was gently filled with 25 ml M medium. GYEP and M media were connected by a bridge over the separation of the two compartments that allowed fungal hyphae to cross from one compartment to the other. Approximately 2 cm^2^ of G. *irregulare* (isolates DAOM-197198 and DAOM-234328) and transformed carrot root co-cultures were individually transferred into each compartment containing M medium. Because AMF grow slowly, plates were incubated at 25°C for 4 weeks until the AMF hyphae grew to reach the bridge. The cultures were examined weekly and carrot roots were trimmed aseptically to prevent their growth into the distal compartment. Controls consisted of Ri T-DNA transformed carrot without AMF and *F. sambucinum* alone (neither roots nor AMF). An agar disk of 0.5 cm diameter containing *F. sambucinum* strain T5 was used to inoculate the distal compartment containing GYEP agar medium on the side opposite *G. irregulare*. Additional controls were performed used a disk of the *F. sambucinum* adjacent to M medium alone (Fs+M) or with carrot-roots not inoculated with *G. irregulare* (Fs+Cr). Each combination of *F. sambucinum*/*G. irregulare* and controls was replicated 20 times and plates were randomly placed in the dark and incubated at 25°C. The growth rate of *F. sambucinum* was checked every 2 days and pictures were recorded to measure the growth area using Image J software available at (http://rsbweb.nih.gov/ij/). Results are reported as means of *F. sambucinum* growth alone on M medium (Fs+M), in the presence of *G. irregulare* (*Gi197198*), or with non-inoculated carrot roots (Fs+Cr).

### RNA isolation and real-time qRT-PCR assays

Total RNA was extracted from the mycelia of three biological replicates of *F. sambucinum*. Fungal material was prepared as described in the DNA extraction section. Total RNA was isolated using TRIZOL Reagent according to the manufacture's instructions. cDNA libraries were constructed by RevertAid 270 H Minus M-MuLV kit (Fermentas) according to the manufacture's instructions. Real-time (RT) PCR reactions were performed in a volume of 10 µl containing 2 µl water, 1 µl of each primer, 1 µl cDNA and 5 µl CYBR green Maxima SYBR Green/ROX qPCR Master Mix. A liquid handling Workstation ep*Motion* 5070 (Eppendorf) was used to optimize RT-PCR assays with small reaction volumes. All genes were run in triplicate on each plate and 3 biological replicates of each treatment were performed. A negative control using Mili-Q water was prepared for each sample. RT-PCR was run on EP RealPlex MasterCycler (Eppendorf) using the following conditions: an initial denaturation step at 95°C for 10 min followed by 39 cycles of 95°C for 15 sec, 60°C for 45 sec (annealing and extension). A final extension was carried out by 95°C for 15 sec followed by 60°C for 1 min. A melting curve was performed from 55 to 95°C with a 0.2°C reading interval. We used two house-keeping genes, ß-tubulin elongation factor EF1α, in our RT-PCR assays. All samples had only a single peak, indicating a pure RT-PCR product and no contamination or primer dimer formation. Data analysis was performed on REST 2009 Software available at http://www.gene-quantification.de/rest-2009.htm as described below in in statistical analysis section.

### Statistical analysis

Analysis of variance was used to examine the significant difference on effects of the AMF *G. irregulare* isolate DAOM-197198 on growth of *F. sambucinum* and Shannon diversity indices between different treatments, and post-hoc comparisons between the treatments were done by the Tukey test using SPSS software v. 17 (SPSS Inc., Chicago, Illinois).

We used Relative Expression Software Tool (REST) for group-wise comparison and statistical analysis of relative expression results as described in Pfaffl et al., [34]. The relative expression ratio of a target gene is computed, based on its real-time PCR efficiencies and the crossing point (CP) difference of an unknown sample versus a control. The purpose of this test is to determine whether there is a significant difference between samples and controls, while taking into account issues of reaction efficiency and reference gene normalization. We used the hypothesis test *P(H_1_)* that represents the probability of the alternate hypothesis that the difference between sample and control groups is due only to chance. The hypothesis test performs a least 2000 times of random reallocations of samples and controls between the groups. Statistical difference are significant when *p*<0.05.

## Supporting Information

Figure S1 **Proposed biosynthetic pathway for 4,15- diacetoxyscirpenol (DAS).**
(TIF)

Figure S2 **Artificial inoculation of potato plants with** ***F. sambucinum*** **strain T5.** (A) Potato plant infected with *F. sambucinum* (right) and non-infected plants (left). (B) Potato tubers harvested from pots infested with *F. sambucinum*.
(TIFF)
