# Supplementary figures and images for: A Fungal Symbiont of Plant-Roots Modulates Mycotoxin Gene Expression in the Pathogen Fusarium sambucinum
Source: PLoS One. 2011 Mar 24;6(3):e17990. doi: 10.1371/journal.pone.0017990 (PMC3063790; doi:10.1371/journal.pone.0017990)

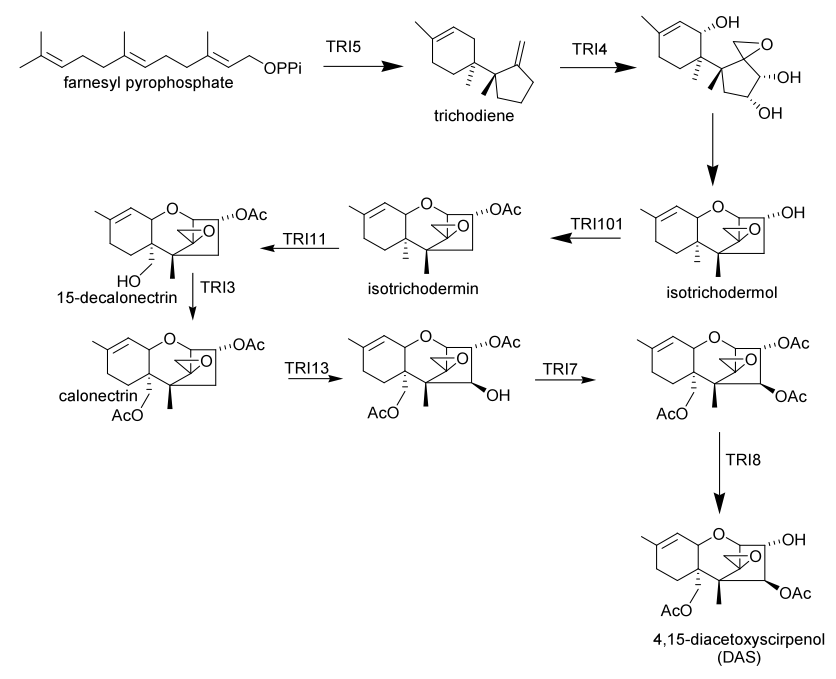

Supplement: Figure S1 — Proposed biosynthetic pathway for 4,15- diacetoxyscirpenol (DAS). (TIF) [file pone.0017990.s001.tif]

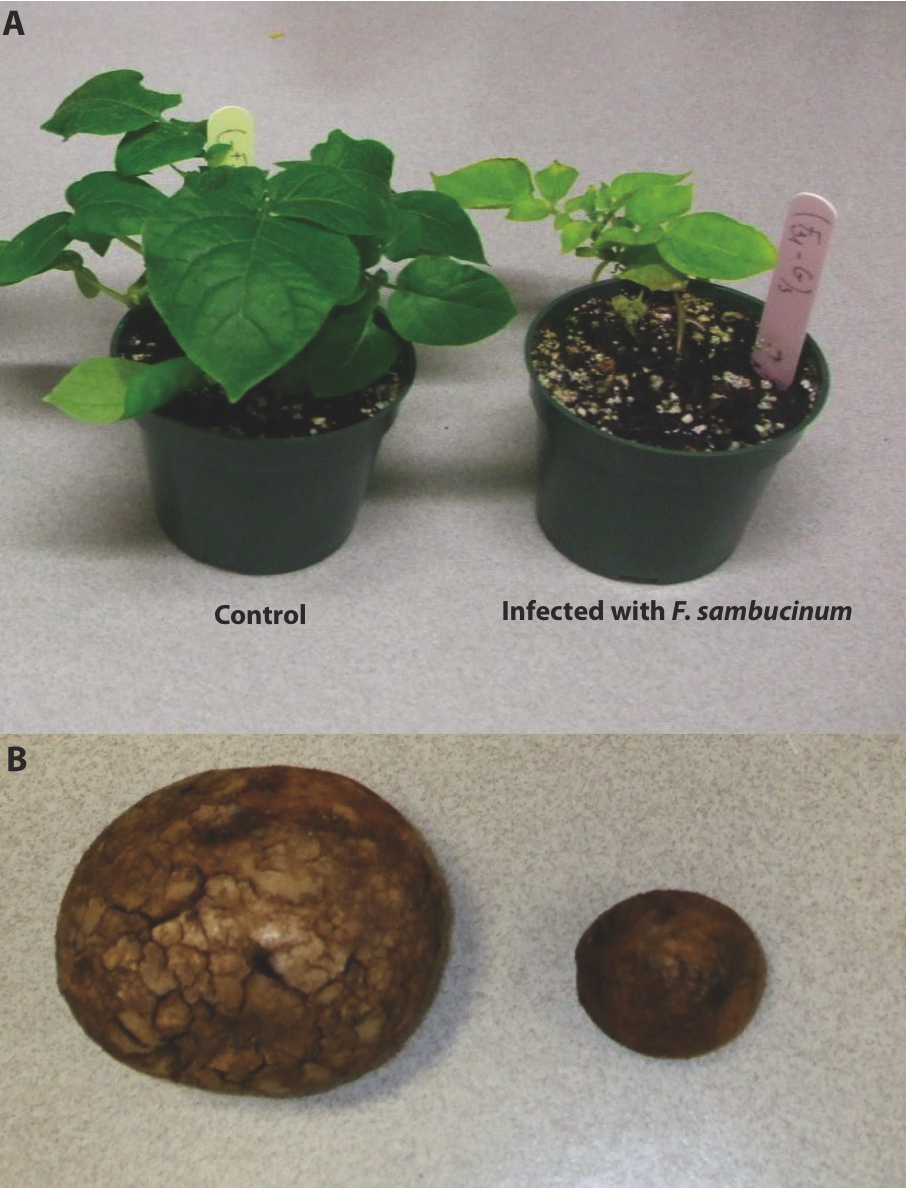

Supplement: Figure S2 — Artificial inoculation of potato plants with F. sambucinum strain T5. (A) Potato plant infected with F. sambucinum (right) and non-infected plants (left). (B) Potato tubers harvested from pots infested with F. sambucinum. (TIFF) [file pone.0017990.s002.tif]
